# Accuracy of the international growth charts to diagnose obesity according to the body composition analysis in US children and adolescents

**DOI:** 10.1017/S0007114524002113

**Published:** 2024-10-14

**Authors:** Mariane Helen de Oliveira, Camila Medeiros da Silva Mazzeti, Joana Araújo, Milton Severo, Débora Borges dos Santos Pereira, Wolney Lisboa Conde

**Affiliations:** 1 School of Social Work, Boston College, Chestnut Hill, MA, USA; 2 Chronic Conditions and Diet Observatory (OCCA), Faculty of Pharmaceutical Sciences, Food and Nutrition (FACFAN), Federal University of Mato Grosso do Sul, Campo Grande, Mato Grosso do Sul, Brazil; 3 EPIUnit - Instituto de Saúde Pública da Universidade do Porto, Porto, Portugal; 4 Laboratório para a Investigação Integrativa e Translacional em Saúde Populacional (ITR), Universidade do Porto Instituto de Saúde Pública, Porto, Portugal; 5 Departmento de Ciências da Saúde Pública e Forenses, e Educação Médica, Faculdade de Medicina da Universidade do Porto, Porto, Portugal; 6 Departamento de Ensino Pré-Graduado, Instituto de Ciências Biomédicas Abel Salazar da Universidade do Porto, Porto, Portugal; 7 Center for Epidemiological Research in Nutrition and Health, Department of Preventive Medicine, School of Medicine, University of São Paulo, São Paulo, Brazil; 8 Department of Nutrition, School of Public Health, University of São Paulo, São Paulo, Brazil

**Keywords:** Child, Adolescent, Nutrition assessment, Obesity, Body composition

## Abstract

This study verified the accuracy of the international BMI references and the allometric BMI reference to diagnose obesity in children and adolescents from the USA. Data from 17 313 subjects were obtained from the National Health and Nutrition Examination Survey between the years 1999–2006 and 2011–2018. Fat Mass Index, Allometric Fat Mass Index and fat mass/fat-free mass were calculated. Receiver operating characteristic curve, AUC, sensitivity, specificity, positive likelihood ratio and negative likelihood ratio were estimated to evaluate the accuracy of the growth references for diagnosing obesity. The International Obesity Task Force, MULT BMI 17 years, MULT BMI 18 years and allometric BMI 19 years achieved the best sensitivity-specificity trade-off for boys, with sensitivities ranging from 0·92 to 0·96 and specificities of 0·94, with positive likelihood ratio of 15·51, 16·17, 13·46 and 18·01, respectively. The negative likelihood ratios were notably low, ranging from 0·04 to 0·08. In girls, the International Obesity Task Force, MULT BMI 17 years and MULT allometric BMI 17 years also demonstrated high sensitivity (0·95–0·97) and specificity (0·92), with positive likelihood ratio values of 11·54, 11·82 and 11·77, respectively and low negative likelihood ratio values (0·03–0·05). In summary, these international growth references presented satisfactory performance to diagnose obesity. However, the MULT growth reference performed better, and the MULT allometric BMI was the only indicator capable of detecting that girls have a higher proportion of fat mass than boys for the same index values. These findings suggest that the MULT growth reference may be a better tool to assess the nutritional status of children and adolescents internationally.

Monitoring body fat mass (FM) during childhood and adolescence is an important way to identify possible health and nutritional risks and to predict them into adulthood^(1,2)^. Body composition analysis is used to detect excessive body FM and excessive body mass^(3,4)^. Among the existing methods for this evaluation, dual-energy X-ray absorptiometry (DXA), developed by Mazess *et al.* ^(5)^, is considered a reference for estimating body composition and has been used to validate equations based on anthropometric measurements^(3,4)^. It presents a good accuracy for predicting body FM, which is associated with cardiometabolic outcomes; however, it is an expensive method and therefore not used on a large scale in population studies^(5,6)^.

Body Mass Index (BMI) is the main tool for monitoring children and adolescent obesity at a population level because it is not an invasive procedure, is easily applicable and does not require expensive equipment^(2,7,8)^. Different BMI growth charts proposed by several institutions for international use were constructed over the years^(7,8)^. These growth charts have been developed using longitudinal or cross-sectional data, with national or international samples and with different inclusion criteria^(7–9)^.

For instance, the growth references from the Centers for Disease Control and Prevention (CDC, 2000)^(10)^ and the WHO (2007)^(11)^ (for children over 5 years old) were constructed based on the population of the USA, while the ones from the WHO (2006)^(12)^, the International Obesity Task Force (IOTF, 2012)^(13,14)^ and the MULT (2023)^(15)^ were constructed based on diverse populations around the world. These methodological divergences in the construction of these international growth charts include the composition of the population and the modelling of the descriptive parameters of the anthropometric index and the cut-off points^(7,8,16)^. These differences generate effects on the nutritional classification and make diagnosing and comparing prevalence difficult^(7,8,16)^.

Considering the effects on the nutritional status of children and adolescents, the MULT growth reference has recently been released^(15,17,18)^. This growth reference was constructed using longitudinal data from ten countries (England, Scotland, Wales, Northern Ireland, Ethiopia, India, Peru, Vietnam, Brazil and Portugal), presenting a multi-ethnic sample^(15,17,18)^. Notably, the MULT growth reference^(15,17,19)^ demonstrates a high degree of concordance with the 2006 WHO growth standard^(12)^ and the IOTF growth reference^(13,14)^, which were also based on multi-ethnic samples^(19)^. The MULT growth reference includes growth charts for height-for-age and BMI-for-age, and it introduces a new approach with the allometric BMI (ABMI) for age^(15,17,18)^.

The ABMI growth charts were developed using the formula weight/height^pt^ proposed by Ben^(20)^, along with the p_t_ exponents specified by age and sex as outlined by Mazzeti *et al.* ^(21)^. These p_t_ exponents were estimated using cross-sectional data from five countries – Brazil, the USA, Mexico, South Korea and England – and were validated through DXA^(21)^. This approach of adjusting the exponents over time was proposed to more accurately estimate the relationship between weight and height during development, particularly during puberty when there is an increase in growth velocity^(18,21)^.

Despite the widespread use of growth references from the CDC^(10)^, WHO^(11)^ and IOTF^(13,14)^ as tools for assessing obesity internationally, a systematic review has indicated that they may not be accurate for several populations^(7)^. Additionally, studies pointed out that the standard BMI formula may not adequately capture the complexities of growth velocity and changes in body composition in children and adolescents^(21,22)^. This limitation arises because, during certain developmental stages, the relationship between weight and height cannot be accurately represented by weight divided by squared height^(21,22)^. Therefore, the aim of this study was to verify the accuracy of the CDC^(10)^, WHO^(11)^, IOTF^(13,14)^ and MULT^(15)^ BMI references and of the MULT ABMI reference^(18)^ to diagnose obesity in US schoolchildren and adolescents according to their body FM estimated by DXA.

## Subjects and methods

### Study design and population

Data from 20 824 children and adolescents from 8 to 20 years old were obtained from the National Health and Nutrition Examination Survey (NHANES) between the years 1999–2006 and 2011–2018^(23–26)^. The NHANES of 2007–2008 and 2009–2010 were not included because they did not have the whole-body DXA variable available^(23–25)^. NHANES is an ongoing study coordinated by the National Center for Health Statistics (which aims to evaluate the health and nutritional status of the non-institutionalised US population over the years^(23–26)^. NHANES evaluates a US representative sample of about 5000 people per year, and its survey includes demographic, socio-economic, dietary and health-related questions, physiological measurements, body composition examination and laboratory tests^(23–26)^.

The anthropometric and body composition measurements of NHANES were collected by trained professionals who followed a standardised examination protocol to ensure data quality^(23–25,27)^. All subjects were measured without personal belongings that could interfere with the anthropometric and DXA assessments^(27–29)^. The analysis of the body composition (whole body) was performed according to the procedures recommended by the manufacturer of the equipment, which was the Hologic Discovery A^(28,29)^. Furthermore, to certify the accuracy of the body composition estimated by this equipment, the Radiology Bone Density Group from the University of California, San Francisco, reviewed and analysed its results using industry-standard techniques^(28,29)^.

NHANES was conducted according to the guidelines laid down in the Declaration of Helsinki, and all procedures involving human subjects were approved by the National Center for Health Statistics Research Ethics Review Board^(30)^. Written informed consent was obtained from all participants aged 18+ years old, and a parent or guardian signed the permission for minors^(23–25,31)^. Additionally, children aged 7–17 years old also provided documented consent and interpreters assisted participants who did not speak or read English or Spanish^(23–25)^. The details of the surveys, as well as their approval from the Research Ethics Committees, have been described in their study protocols and in previous studies^(23–25,29–31)^.

**Fig. 1.**
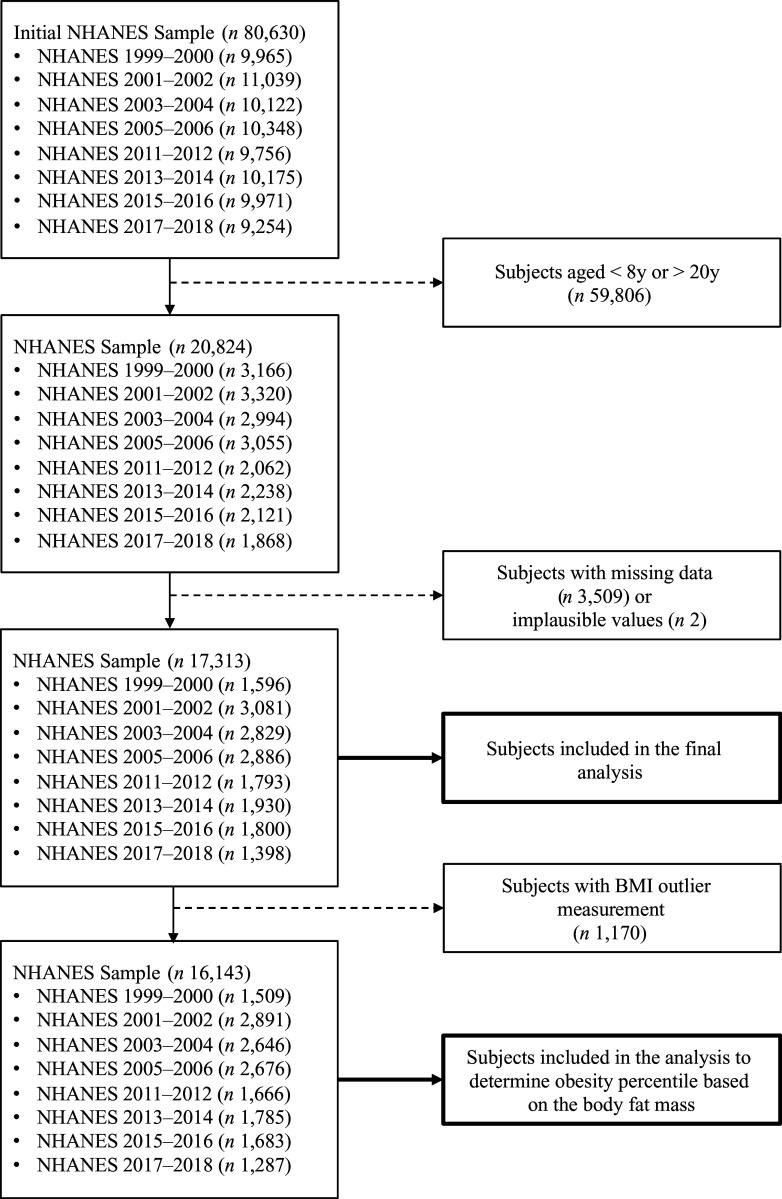
Flow chart of the subject selection. NHANES, National Health and Nutrition Examination Survey.

The NHANES (1999–2006/2011–2018) provided data from 80 630 subjects^(23–26)^. For the analysis of this study, we selected demographic (sex, age, ethnicity), body measurements (weight, height) and body composition (FM, fat-free mass (FFM)) data of subjects aged up to 20 years old. Moreover, as shown in Fig. 1, we limited our study to subjects aged between 8 and 20 years old because there was no DXA examination for children younger than 8 years old. In the data processing, we excluded 3509 subjects who had missing data (demographic, anthropometric or body composition data) and two subjects with implausible BMI values, determined as a BMI-for-age *z*-score below – 5 sd or above 5 sd ^(32,33)^. This exclusion was performed based on 2007 WHO BMI reference values, as it is a worldwide well-known criterion validated by several studies^(11,32–36)^.

### Data processing and analysis

In order to obtain a reference population sample to estimate the percentiles for obesity based on the FM estimated by the DXA, we removed subjects with outlier BMI values. Outlier values were determined as BMI-for-age *z*-score below – 3 sd or above + 3 sd according to the WHO reference values^(11,32,33)^. This approach was employed following the recommendations of the WHO (2006) for constructing growth charts and estimating cut-offs^(12,33)^. According to these guidelines, unhealthy weight data should be removed to avoid skewing the growth reference, and ideally, obesity should affect only 3 % of a healthy population^(12,33)^. This is particularly important given that US national data indicates that in 2017–2018, nearly one in five children and adolescents aged 2–19 years (19·3 %) had obesity^(37)^. Such trends underscore the necessity of removing extreme values, especially when estimating cut-offs using data from countries with high obesity rates^(12)^.

Concerning the obesity classification, the FM was used to calculate a Fat Mass Index (FMI), as proposed by Kelly *et al.* ^(29)^, and an Allometric Fat Mass Index (AFMI) as proposed by us. The FMI is the ratio of FM (kg) to squared height (m^2^), following the BMI concept but using FM instead of body mass (weight). The AFMI is the ratio of FM (kg) to height (m) raised to the p_t_ exponents^(29)^. These exponents, ranging from 1·9 to 3·1 and varying by age and sex, were previously estimated by Mazzeti *et al.* ^(21)^ and adapted by De Oliveira *et al.* in the ABMI growth reference^(18)^.

The obesity classification based on the body fat was determined as FMI or AFMI percentile ≥ 95^th^, while the obesity classification of the BMI and ABMI were assessed according to the values proposed by the CDC^(10)^, WHO^(11)^, IOTF^(13,14)^ and MULT^(15)^ BMI references and the MULT ABMI reference^(18)^. For both sexes, the lowest obesity percentiles for BMI and ABMI were observed with CDC^(10)^ (95·0^th^) and WHO^(11)^ (97·0^th^). For males, the highest obesity percentile was found with MULT ABMI^(18)^, with a cut-off estimated at 18 years old (99·1^th^). For females, the highest percentiles were observed with MULT BMI^(15)^, with a cut-off estimated at 17 years old (98·6^th^) and with IOTF^(14)^ (98·6^th^), as shown in Table 1. Additionally, the FM/FFM was calculated for each participant, and sex-specific scatter plots for the relation between FMI, AFMI, FM/FFM and the indexes of BMI and ABMI were plotted^(38)^.

**Table 1.** Obesity percentile corresponding to the value of 30 kg/m^2^ at 17–20 years old of the MULT ABMI reference and MULT, CDC, WHO and IOTF BMI references for boys and girls

	Reference	Boys	Girls
17 years	MULT ABMI^(18)^	–	98·3th
	MULT BMI^(15)^	98·7th	98·6th
18 years	MULT ABMI^(18)^	99·1th	97·9th
	MULT BMI^(15)^	98·3th	98·1th
	IOTF^(14)^	98·9th	98·6th
19 years	MULT ABMI^(18)^	98·4th	97·8th
	MULT BMI^(15)^	97·8th	97·5th
	WHO^(11)^	97·0th	97·0th
20 years	MULT ABMI^(18)^	97·6th	97·8th
	MULT BMI^(15)^	97·2th	96·9th
	CDC^(10)^	95·0th	95·0th

ABMI, allometric BMI; IOTF, International Obesity Task Force; CDC, Centers for Disease Control and Prevention.

Moreover, to evaluate accuracy of the growth references, the *z*-score of each growth chart was calculated using the L (skewness), M (median) and S (CV) values. Subsequently, these scores were converted into percentiles, and the obesity classification was determined based on the percentile cut-offs of the CDC^(10)^, WHO^(11)^, IOTF^(14)^, MULT BMI^(15)^ and MULT ABMI^(18)^. The diagnostic accuracy, defined as the proportion of all tests that give a correct result, was performed for the CDC^(10)^, WHO^(11)^, IOTF^(14)^ and MULT^(15)^ BMI growth references, according to the obesity diagnostic criteria classified by the FMI^(29,39)^. For the MULT ABMI^(18)^, the AFMI was used instead.

Furthermore, sensitivity, specificity and positive likelihood ratio and negative likelihood ratio were calculated, specified by sex^(39)^. The positive likelihood ratio indicates how much more likely subjects who test positive are to actually have the condition compared with those who test negative^(39)^. The negative likelihood ratio, on the other hand, measures how much less likely a negative test result is to occur in individuals with the condition compared with those without it^(39)^. Additionally, the receiver operating characteristic curve and its AUC were calculated and plotted to provide a measure of discrimination and allow comparison among the growth references^(40)^. The optimal cut-off for obesity percentiles was estimated for each growth reference through receiver operating characteristic curve analysis using the pROC package^(40,41)^. This optimal cut-off is the threshold that maximises the distance from the identity (diagonal) line, with the optimality criterion being the maximum of the sum of sensitivities and specificities^(40,41)^. All statistical analyses were performed using R software version 4·2·1 for Windows^(42)^.

## Results

After the exclusion of subjects with missing data or implausible values, data from 17 313 subjects (54·5 % males) remained for the diagnostic analysis, as shown in Fig. 1. For the obesity classification based on the FMI and AFMI, after removing subjects with severe obesity or underweight, 16 143 subjects (54·2 % males) were selected, as presented in Fig. 1. The largest ethnic group of our sample was non-Hispanic Black (29·24 %), followed by Mexican American (28·3 %), non-Hispanic White (26·7 %), other races (including multi-racial) (9·3 %) and other Hispanic (6·5 %).

**Fig. 2.**
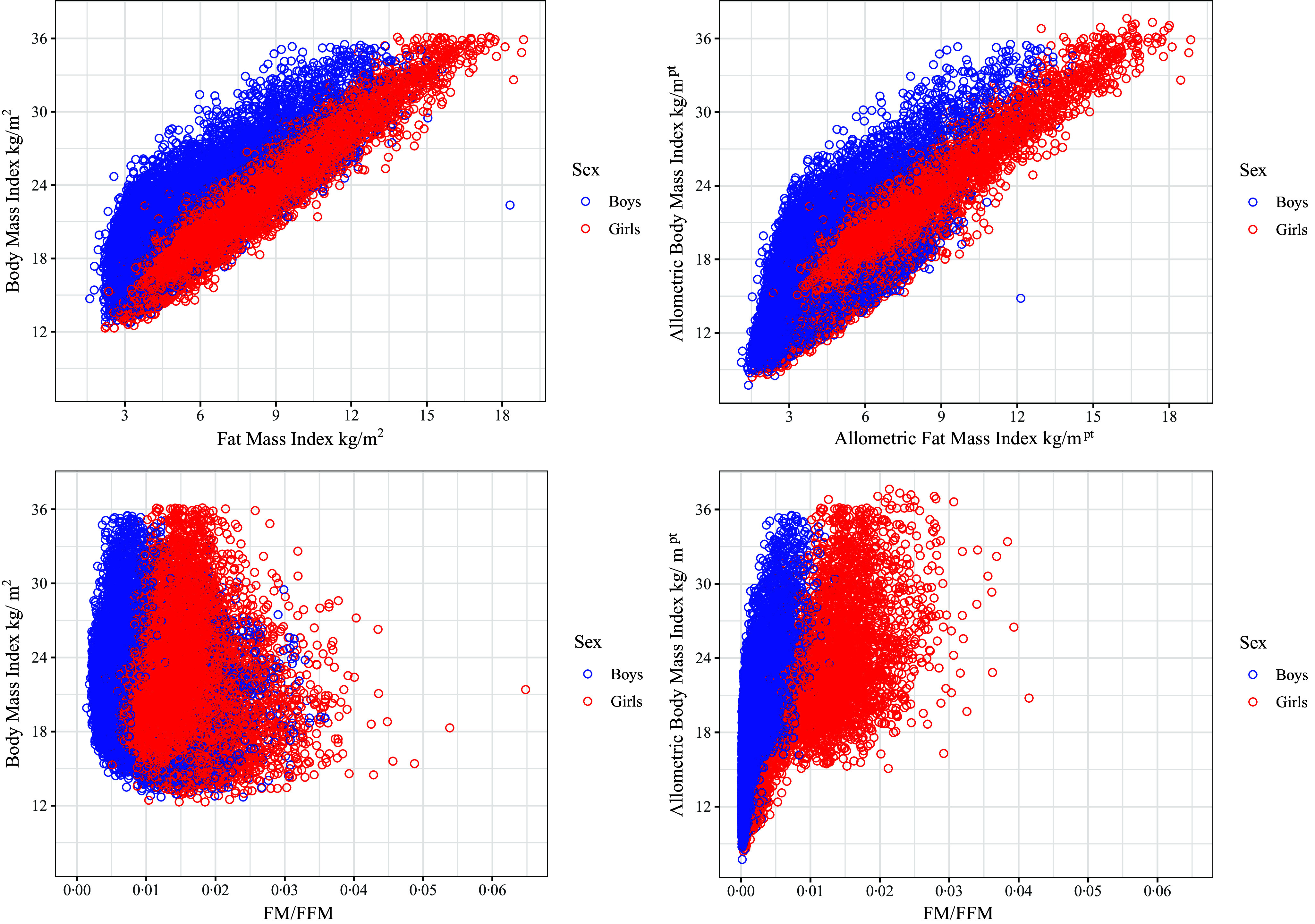
Scatter plots of the Fat Mass Index and BMI, Allometric Fat Mass Index and allometric BMI (ABMI), fat mass/fat-free mass (FM/FFM) and BMI and FM/FFM and ABMI.

In this study, both BMI and ABMI were associated with FM, with the ABMI presenting greater dispersion compared with BMI. There was a strong positive correlation between BMI and FMI and between ABMI and AFMI, as shown in Fig. 2. Moreover, the analyses with the FM/FFM pointed out that this index is associated with both BMI and ABMI. Notably, in the scatter plot between ABMI and FM/FFM (Fig. 2), there is a clear distinction between sexes, indicating that girls have more FM than FFM in their body composition than boys for the same ABMI values.

**Fig. 3.**
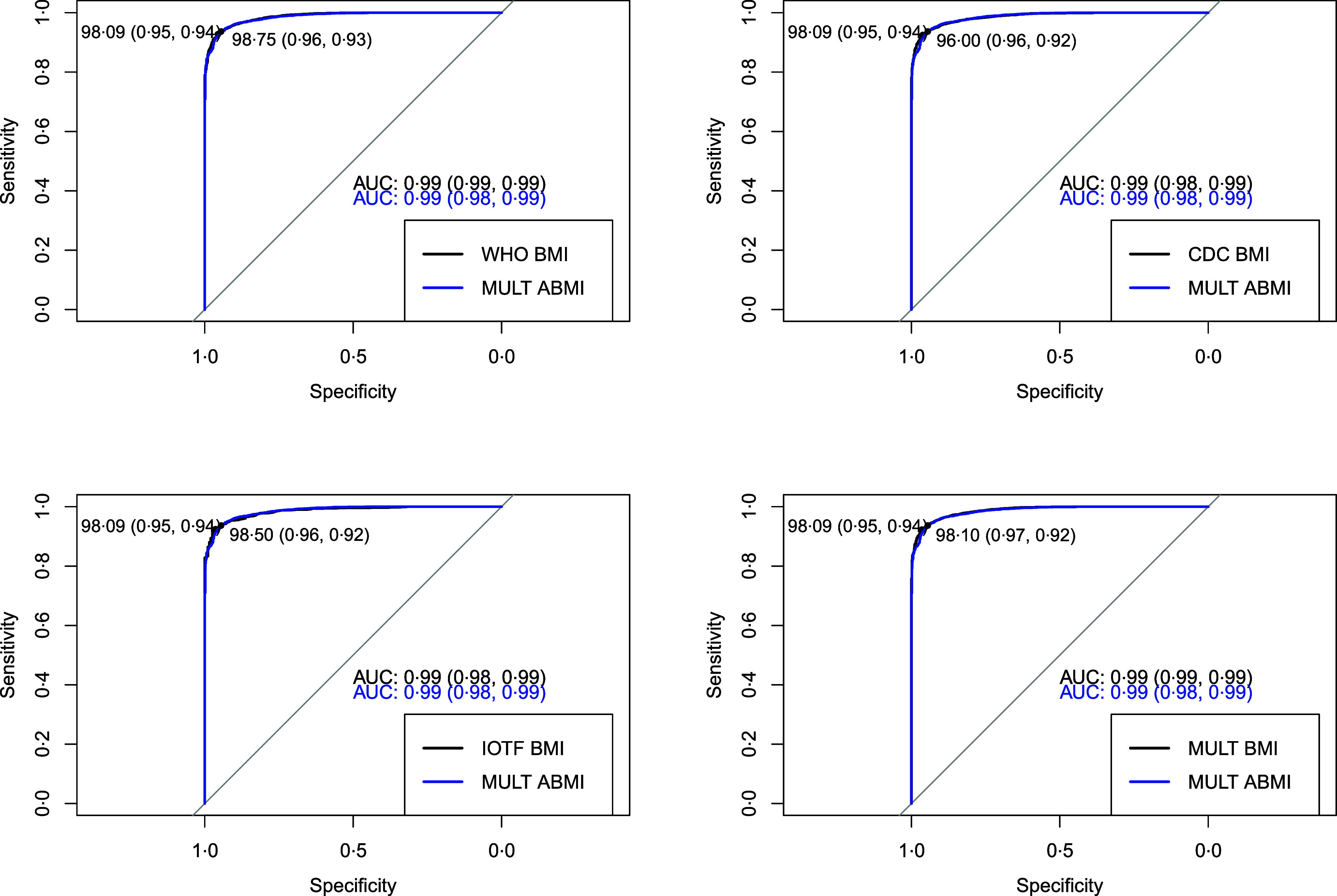
Receiver operating characteristic curve and AUC comparison among the four BMI references and the allometric BMI reference to diagnose obesity in boys according to the body fat mass 95^th^ percentile.

**Fig. 4.**
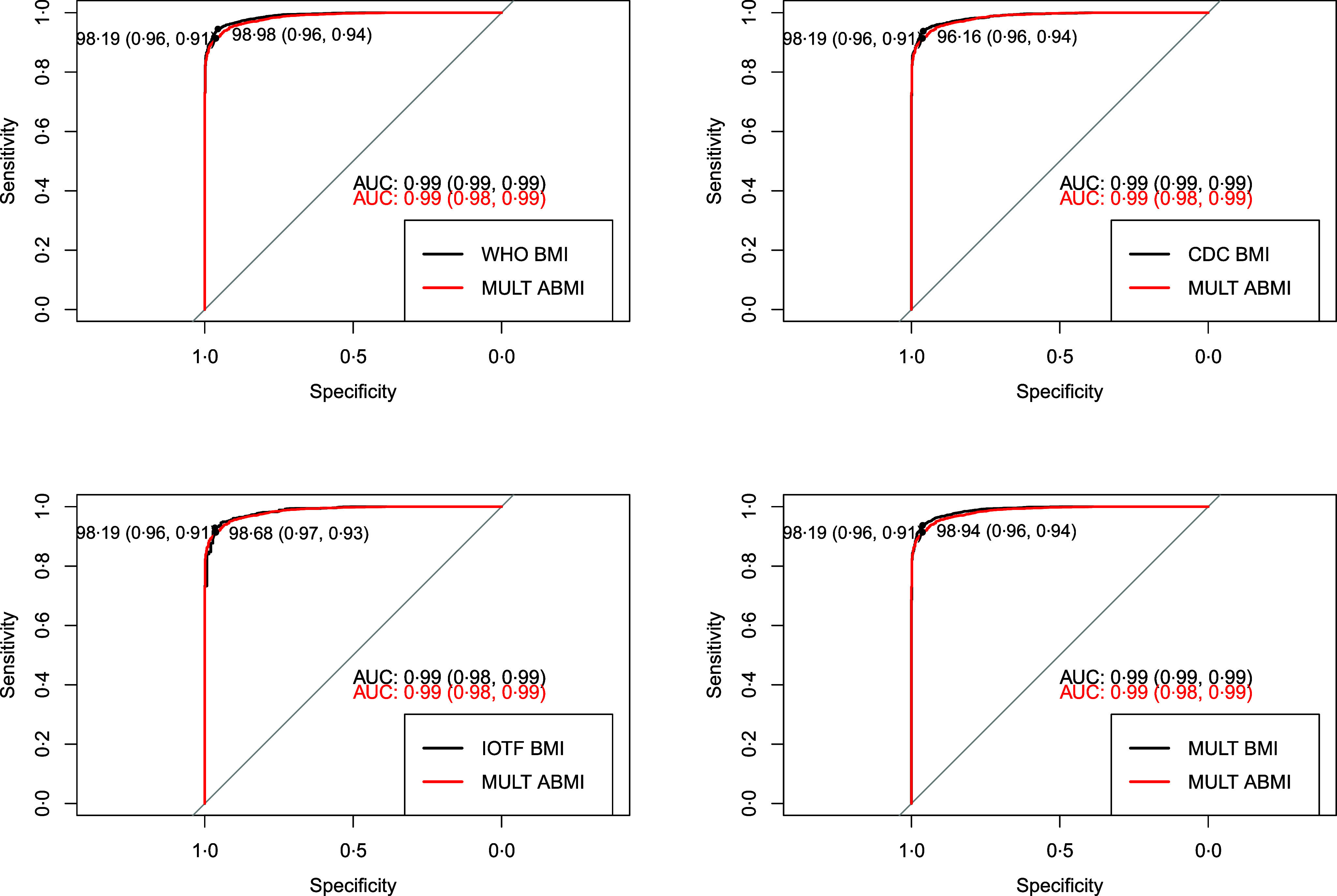
Receiver operating characteristic curve and AUC comparison among the four BMI references and the allometric BMI reference to diagnose obesity in girls according to the body fat mass 95^th^ percentile.

The comparison between the BMI and the ABMI references through the body composition analysis performed by the receiver operating characteristic curve and the AUC analysis are presented for boys and girls, respectively, in Figs. 3 and 4. Regarding the optimal cut-off for obesity in the MULT BMI reference^(15)^ (boys), it is at the 98·1^th^ percentile, which is similar to the obesity percentiles estimated at 18 years (98·3^th^ percentile) and 19 years (97·8^th^ percentile). For girls, the optimal cut-off in the MULT BMI reference^(15)^ is at the 98·9^th^ percentile, which closely aligns with the obesity percentile estimated at 17 years (98·6^th^ percentile). Similar results were found for the MULT ABMI reference^(18)^, the closest values to the optimal obesity percentile cut-offs (boys = 98·1^th^ | girls = 98·2^th^) were at 19 years old (98·4^th^ percentile) for boys and 18 years old (97·9^th^ percentile) for girls.

The FMI and AFMI values corresponding to the 95^th^ percentile, as well as the p_t_ exponents per age and sex applied in the ABMI reference^(18)^, are presented in Table 2. The AFMI presented lower cut-off values than FMI for males from 5 years old to 18 years old and for females from 5 years old to 15 years old. In these age ranges, the exponents in the ABMI were higher than 2. The opposite occurred for females aged 15 years, and the AFMI values were higher than the ones from FMI, having the ABMI exponent lower than 2.

**Table 2.** FMI and AFMI cut-off points for obesity (95^th^ percentile) and exponent values applied in the ABMI reference specified by age (years) and sex

	Boys			Girls		
Age (years)	FMI	AFMI	p_t_	FMI	AFMI	p_t_
8·0	7·3	5·9	2·8	9·0	7·0	2·8
8·5	7·7	6·1	2·8	7·0	5·7	2·8
9·0	8·2	6·3	2·9	9·2	6·9	3·0
9·5	8·3	5·8	2·9	9·5	7·0	3·0
10·0	10·3	7·2	3·0	11·2	7·6	3·1
10·5	9·8	7·0	3·0	12·9	8·2	3·1
11·0	11·5	7·5	3·1	11·8	7·8	3·0
11·5	10·4	6·9	3·1	10·9	7·2	3·0
12·0	10·3	6·9	3·0	11·3	7·5	2·8
12·5	11·1	7·1	3·0	12·1	8·6	2·8
13·0	10·3	6·6	2·9	13·9	11·1	2·5
13·5	10·9	7·5	2·9	11·0	8·7	2·5
14·0	9·8	7·0	2·7	13·0	11·8	2·2
14·5	12·0	8·4	2·7	12·9	11·8	2·2
15·0	10·8	8·3	2·5	13·0	13·6	1·9
15·5	10·5	7·9	2·5	13·4	14·0	1·9
16·0	11·1	9·4	2·3	14·1	14·1	2·0
16·5	11·2	9·5	2·3	13·7	13·7	2·0
17·0	10·7	10·1	2·1	15·3	15·3	2·0
17·5	10·9	10·3	2·1	13·0	13·0	2·0
18·0	9·4	9·4	2·0	15·5	15·5	2·0
18·5	9·4	9·4	2·0	13·9	13·9	2·0
19·0	9·7	9·7	2·0	15·4	15·4	2·0
19·5	12·0	12·0	2·0	14·8	14·8	2·0
20·0	12·2	12·2	2·0	13·7	13·7	2·0

FMI, Fat Mass Index (FM/height^2^); AFMI, Allometric Fat Mass Index (FM/height^pt^).

p_t_: exponents per age estimated previously by Mazzeti *et al.*^(21)^ and adapted by De Oliveira *et al.*^(18)^.

In terms of diagnostic accuracy for boys, the IOTF^(14)^, MULT BMI 17 years^(15)^, MULT BMI 18 years^(15)^ and ABMI 19 years^(18)^ achieved the best sensitivity-specificity trade-off, as shown in Table 3. The IOTF^(14)^ demonstrated a sensitivity of 0·94 (95 % CI 0·92, 0·95) and specificity of 0·94 (95 % CI 0·93, 0·94). Similarly, MULT BMI 17 years^(15)^ also showed a sensitivity of 0·94 (95 % CI 0·92, 0·95) and specificity of 0·94 (95 % CI 0·94, 0·95). MULT BMI 18 years^(15)^ achieved a sensitivity of 0·96 (95 % CI 0·95, 0·97) and specificity of 0·93 (95 % CI 0·92, 0·93), while ABMI 19 years^(18)^ had a sensitivity of 0·92 (95 % CI 0·91, 0·94) and specificity of 0·95 (95 % CI 0·94, 0·95). These results are reflected in their positive likelihood ratio, with IOTF^(14)^ at 15·51 (95 % CI 14·23, 16·90), MULT BMI 17 years^(15)^ at 16·17 (95 % CI 14·81, 17·66), MULT BMI 18 years^(15)^ at 13·46 (95 % CI 12·44, 14·56) and ABMI 19 years^(18)^ at 18·01 (95 % CI 16·40, 19·79). The negative likelihood ratios were also noteworthy, with IOTF^(14)^ at 0·07 (95 % CI 0·05, 0·08), MULT BMI 17 years^(15)^ at 0·07 (95 % CI 0·05, 0·08), MULT BMI 18 years^(15)^ at 0·04 (95 % CI 0·03, 0·06) and ABMI 19 years^(18)^ at 0·08 (95 % CI 0·07, 0·10).

**Table 3.** Sensitivity, specificity, diagnostic accuracy, LR+, LR– of the four BMI references and of the ABMI reference according to the body FM

Boys (*n* 9,432)	Sensitivity	95 % CI	Specificity	95 % CI	LR+	95 % CI	LR–	95 % CI	Diagnostic accuracy	95 % CI
WHO^(11)^	0·98	0·97, 0·99	0·89	0·88, 0·89	8·58	8·07, 9·11	0·02	0·02, 0·03	0·90	0·89, 0·90
CDC^(10)^	0·98	0·97, 0·99	0·89	0·88, 0·89	8·78	8·26, 9·34	0·02	0·01, 0·03	0·90	0·89, 0·91
IOTF^(14)^	0·94	0·92, 0·95	0·94	0·93, 0·94	15·51	14·23, 16·90	0·07	0·05, 0·08	0·94	0·93, 0·94
MULT BMI 17 years^(15)^	0·94	0·92, 0·95	0·94	0·94, 0·95	16·17	14·81, 17·66	0·07	0·05, 0·08	0·94	0·94, 0·95
MULT BMI 18 years^(15)^	0·96	0·95, 0·97	0·93	0·92, 0·93	13·46	12·44, 14·56	0·04	0·03, 0·06	0·93	0·93, 0·94
MULT BMI 19 years^(15)^	0·97	0·96, 0·98	0·91	0·90, 0·92	10·77	10·05, 11·54	0·03	0·02, 0·04	0·92	0·91, 0·92
MULT BMI 20 years^(15)^	0·98	0·97, 0·99	0·89	0·89, 0·90	9·10	8·55, 9·68	0·02	0·01, 0·03	0·90	0·90, 0·91
MULT ABMI 18 years^(18)^	0·85	0·83, 0·87	0·97	0·96, 0·97	27·12	24·00, 30·64	0·15	0·13, 0·18	0·95	0·95, 0·96
MULT ABMI 19 years^(18)^	0·92	0·91, 0·94	0·95	0·94, 0·95	18·01	16·40, 19·79	0·08	0·07, 0·10	0·95	0·94, 0·95
MULT ABMI 20 years^(18)^	0·96	0·95, 0·97	0·92	0·91, 0·93	11·91	11·06, 12·82	0·04	0·03, 0·06	0·92	0·92, 0·93
**Girls (** ***n*** **7,881)**	Sensitivity	95 % CI	Specificity	95 % CI	LR+	95 % CI	LR–	95 % CI	Diagnostic accuracy	95 % CI
WHO^(11)^	0·99	0·98, 0·99	0·88	0·88, 0·89	8·49	7·96, 9·06	0·01	0·01, 0·02	0·90	0·89, 0·90
CDC^(10)^	0·99	0·98, 0·99	0·88	0·88, 0·89	8·51	7·98, 9·09	0·02	0·01, 0·03	0·90	0·89, 0·90
IOTF^(14)^	0·97	0·96, 0·98	0·92	0·91, 0·92	11·54	10·67, 12·48	0·03	0·02, 0·04	0·92	0·92, 0·93
MULT BMI 17 years^(15)^	0·97	0·96, 0·98	0·92	0·91, 0·92	11·82	10·92, 12·80	0·03	0·02, 0·04	0·92	0·92, 0·93
MULT BMI 18 years^(15)^	0·98	0·97, 0·99	0·90	0·89, 0·91	9·72	9·06, 10·43	0·02	0·01, 0·03	0·91	0·90, 0·91
MULT BMI 19 years^(15)^	0·99	0·98, 0·99	0·88	0·87, 0·89	8·32	7·80, 8·87	0·02	0·01, 0·03	0·89	0·89, 0·90
MULT BMI 20 years^(15)^	0·99	0·98, 1·00	0·86	0·85, 0·87	7·21	6·80, 7, 65	0·01	0·00, 0·02	0·88	0·87, 0·88
MULT ABMI 17 years^(18)^	0·95	0·94, 0·97	0·92	0·9, 0·93	11·77	10·86, 12·75	0·05	0·04, 0·07	0·91	0·91, 0·92
MULT ABMI 18 years^(18)^	0·96	0·95, 0·98	0·91	0·90, 0·91	10·27	9·54, 11·06	0·04	0·03, 0·05	0·91	0·91, 0·92
MULT ABMI 19 years^(18)^	0·96	0·95, 0·97	0·91	0·90, 0·91	10·29	9·55, 11·08	0·04	0·03, 0·06	0·91	0·91, 0·92
MULT ABMI 20 years^(18)^	0·97	0·96, 0·98	0·90	0·90, 0·91	10·16	9·44, 10·93	0·03	0·02, 0·05	0·91	0·91, 0·92

LR+, positive likelihood ratio; LR–, negative likelihood ratio; CDC, Centers for Disease Control and Prevention; ABMI, allometric BMI; IOTF, International Obesity Task Force.

Diagnostic accuracy: Correctly classified proportion.

17 years: The cut-off point was calculated using the BMI value of 30 kg/m^2^ at 17 years old.

18 years: The cut-off point was calculated using the BMI value of 30 kg/m^2^ at 18 years old.

19 years: The cut-off point was calculated using the BMI value of 30 kg/m^2^ at 19 years old.

20 years: The cut-off point was calculated using the BMI value of 30 kg/m^2^ at 20 years old.

For girls, the diagnostic accuracy of the IOTF^(14)^, MULT BMI 17 years^(15)^ and MULT ABMI 17 years^(18)^ demonstrated the best sensitivity-specificity trade-off, as presented in Table 3. The IOTF^(14)^ achieved a sensitivity of 0·97 (95 % CI 0·96, 0·98) and a specificity of 0·92 (95 % CI 0·91, 0·92). Similarly, MULT BMI 17 years^(15)^ reported a sensitivity of 0·97 (95 % CI 0·96, 0·98) and specificity of 0·92 (95 % CI 0·91, 0·92). MULT ABMI 17 years^(18)^ showed a slightly lower sensitivity of 0·95 (95 % CI 0·94, 0·97) and a specificity of 0·92 (95 % CI 0·92, 0·93). Compared with other growth references such as the CDC^(10)^ and WHO^(11)^, these references exhibited the highest positive likelihood ratio, with IOTF^(14)^ at 11·54 (95 % CI 10·67, 12·48), MULT BMI 17 years^(15)^ at 11·82 (95 % CI 10·92, 12·80) and MULT ABMI 17 years^(18)^ at 11·07 (95 % CI 10·86, 12·75). Additionally, all three references exhibited low negative likelihood ratio, with IOTF^(14)^ at 0·03 (95 % CI 0·02, 0·04), MULT BMI 17 years^(15)^ at 0·03 (95 % CI 0·02, 0·04) and MULT ABMI 17 years^(18)^ at 0·05 (95 % CI 0·04, 0·07).

Additionally, our study pointed out sex differences to establish the obesity cut-off points in the MULT growth references^(15,18)^. The BMI value of 30 kg/m^2^ to diagnose obesity seems to be accurate to be applied at 19 years old in boys, while for girls, it seems to be more adequate to be applied around 17 or 18 years old. These diagnostic accuracy analyses are presented in Table 3.

## Discussion

In our analysis, the BMI and the ABMI showed to be positively associated with adiposity. However, the relationship between body composition and human growth is not yet fully understood^(43)^. It is influenced by environmental, genetic, maternal and dietary factors, especially in early childhood^(43)^. A study involving Iranian children aged 6 years highlighted that maternal-related factors significantly affect a child’s risk of developing obesity^(44)^. These factors include gestational diabetes, high maternal BMI before pregnancy, high gestational weight gain, maternal smoking during pregnancy, paternal smoking, high birth weight and an early introduction of solid foods, all of which increase the odds of developing obesity in preschool children^(44)^.

Considering the variety of international growth charts available and their differing criteria for modelling descriptive growth parameters, the MULT growth reference^(15,17,18)^ appears to be an effective tool for screening the nutritional status of children and adolescents, demonstrating superior performance among international growth references^(19)^. One reason for this is that it was developed using more recent longitudinal data from children who likely reflect the secular trend in height^(17)^. Moreover, MULT was developed with a more ethnically diverse sample, which enhances its applicability to US children and adolescents, given the country’s own ethnic diversity^(15,17,18)^.

Additionally, the application of a dynamic exponent in the ABMI formula^(18,21)^ better adjusts the relationship between weight and height, particularly in boys during puberty, when significant changes in growth velocity and body composition occur due to the development of secondary sex characteristics^(45)^. Some studies pointed out that in this life stage, the exponent 2, which is applied in the BMI formula, does not adjust the body proportions properly, and to have an adequate adjustment, the exponent should increase around the value of 3^(22,46)^. Supporting this, a study involving Iranian children aged 6 years found that, although BMI performed well in identifying general obesity, the tri-ponderal index (weight/height^3^) demonstrated even greater accuracy^(47)^.

Consequently, a study conducted by Zapata *et al.* ^(48)^ with white Spanish subjects aged 6–17 years found that a significant proportion of those classified as normal weight or overweight by BMI actually had a body fat percentage (%) measured by air displacement plethysmography within the obesity range. Subjects not classified as obese by BMI but were classified as such by BF% showed higher levels of cardiometabolic risk markers, including high blood pressure, C-reactive protein, glucose, uric acid, leucocytes count and reduced HDL-cholesterol^(48)^. Additionally, a study conducted with the Swiss population (aged 18 years and over) suggested that BMI is highly influenced by height, as height increases, the probability of an individual being classified in the overweight category (BMI > 25 kg/m^2^) decreases^(49)^. These findings highlight the limitations of using BMI to assess nutritional status in children and adolescents.

In this way, the use of BMI seems to be less accurate for adolescents due to the influence of sexual maturation on body composition^(50)^. Silva *et al.* ^(51)^ highlight that the non-uniform variation in the increase in body mass and height gain throughout adolescence represents a challenge for assessing nutritional status. This phenomenon makes the interpretation of anthropometric changes and body composition more complex, particularly when using one-dimensional indices such as BMI for nutritional assessment^(51)^.

Moreover, the p_t_ exponents of the ABMI are stratified per sex, following the growth patterns and pubertal stage of each sex, which is essential to capture the relation between FM and body proportion among the sexes^(18,21)^. The ABMI analysis showed that, proportionally, girls exhibit a higher ratio of FM to FFM compared with boys at the same index values. These findings are aligned with several studies indicating that girls generally have a higher percentage of body fat than boys^(52,53)^. During puberty, this difference between the sexes is evident in the body composition changes, while girls gain more FM, boys acquire more FFM and skeletal mass^(52,53)^.

In this way, the highest exponent values of the ABMI occurred before in girls than in boys, as it is directly correlated to sexual maturation^(18,21)^. This can explain why the best diagnostic accuracy for obesity in girls is achieved by applying the BMI value of 30 kg/m^2^ at a younger age (17 years old), while for boys, the best diagnostic accuracy is using this cut-off value around 19 years of age. Another possible explanation for that is the increased prevalence of early puberty, especially in countries affected by high rates of childhood obesity, such as the USA^(54,55)^. Studies pointed out that nutritional habits are the main factor for this prevalence increase^(54–56)^. A diet with excessive consumption of processed, high-energy and high-fat food can lead to precocious puberty^(54–56)^.

Regarding the cut-offs for underweight, overweigh and obesity, there is a concern in the scientific community about the values and the proper age to establish them^(13)^. For adults, and to establish the cut-offs in the WHO growth reference^(11)^, the WHO applied the BMI values of 25 kg/m^2^ and 30 kg/m^2^, as cut-off points for overweight and obesity, respectively^(57)^. The IOTF^(13,14)^ also applied the values of 25 kg/m^2^ and 30 kg/m^2^ to derive the cut-off percentile for overweight and obesity, but they applied it at 18 years old, while suggesting the value of 17 kg/m^2^ to estimate underweight. On the other hand, the CDC^(10)^ used *z*-scores and percentiles from the sample to derive their cut-off points around 20 years of age^(58)^.

An advantage of the MULT BMI^(15)^ and ABMI references^(18)^ is being constructed using recent longitudinal data of a multi-ethnic sample and the application of the BMI value of 30 kg/m^2^ to establish the obesity percentile cut-off. This cut-off point is well-known as a risk factor for developing non-communicable diseases^(57)^. Additionally, the MULT references presented high performance to detect obesity in Brazilian and US children and adolescents, which are a multi-ethnic countries, which suggests that the MULT references may fit for international use^(19)^. Another advantage of the MULT references^(15,18)^ is the various obesity percentiles based on the adult BMI cut-off of 30 kg/m^2^ at ages 17–20 years. This flexibility allows countries to assess and compare these options, enabling them to select the cut-off that best aligns with their specific growth patterns. It is important to note that while both the WHO^(11)^ and IOTF^(13,14)^ applied the adult BMI cut-off to estimate overweight and obesity percentiles, they had limitations regarding upper age limits, with IOTF^(13,14)^ capping at 18 years and WHO^(11)^ at 19 years. Consequently, it is not possible to estimate these percentile cut-offs in the same way as with the MULT growth reference^(15,17,18)^.

Furthermore, in the assessment of optimal cut-offs for obesity within the MULT BMI reference^(15)^, a notable distinction emerged between boys and girls. For boys, the values established at 18 and 19 years old closely approached the optimal cut-off, whereas for girls, the optimal value aligned earlier with the cut-off, specifically at 17 years old. This observed trend persisted when examining the MULT ABMI reference^(18)^, with optimal obesity percentile cut-offs for boys corresponding to values at 19 years old, while for girls, it occurred at 17 years old. This observation may be attributed to the earlier onset of sexual maturation in girls compared with boys, leading to the cessation of growth at an earlier stage^(17,45)^. Consequently, it is reasonable to expect that their cut-off point is attained earlier in the developmental timeline. These disparities highlight sex differences in determining the timing of cut-off points, underscoring the need for sex-specific considerations in obesity assessment.

The major strengths of this study are being a representative sample of a multi-ethnic country (USA); the use of anthropometric data gathered by trained professionals, which is supposed to reduce the odds of measurement errors and social desirability bias; and the use of DXA to assess the body composition, which is an advance technique considered as a reference to estimate the FM^(3,23–25,29)^. Nevertheless, there are certain limitations in our study, such as the lack of a reference to determine obesity in children and adolescents according to their body composition, especially their FM. Therefore, we utilised the 95^th^ percentile in our analysis. Additionally, another limitation arises from the unavailability of body composition data for children younger than 8 years old, confining our analysis exclusively to schoolchildren and adolescents.

In summary, the increase in obesity prevalence among children and adolescents underscores the urgent need for effective nutritional surveillance. Enhancing the understanding of obesity in children and adolescents and implementing targeted interventions can reduce obesity’s long-term impact on people’s health and well-being. In this way, this study analysed the accuracy of the international BMI and the ABMI references in diagnosing obesity, with a focus on FM estimated through body composition analysis. It is important to highlight that BMI was not originally designed as a predictor of body fat, even though its cut-offs have been associated with obesity-related diseases, leading to its widespread use as a diagnostic tool for obesity screening^(57,59)^. Compared with the CDC^(10)^, WHO^(11)^ and IOTF^(13,14)^ BMI references, the MULT BMI^(15)^ presented the highest performance. Moreover, the MULT ABMI^(18)^ reference, a new addition to growth charts, showed p_t_ exponents aligned with the pubertal stage. It was the only reference capable of detecting more FM than FFM in girls than boys for the same index values. These findings suggest that the MULT BMI^(15)^ and ABMI^(15)^ references may be a more effective tool than other references for assessing the nutritional status of multi-ethnic children and adolescents.
